# Metabolic syndrome severity score associates with structural brain features and functional connectivity of Default Mode Network subsystems in the HABS‐HD study

**DOI:** 10.1002/alz.091808

**Published:** 2025-01-09

**Authors:** Zachary D. Green, Sid E. O'Bryant, Leigh A. Johnson, Jeffrey M Burns, Jill K Morris

**Affiliations:** ^1^ University of Kansas Medical Center, Kansas City, KS USA; ^2^ University of Kansas Alzheimer's Disease Research Center, Fairway, KS USA; ^3^ University of North Texas Health Science Center, Fort Worth, TX USA

## Abstract

**Background:**

States of altered metabolic health such as metabolic syndrome (MetS), obesity, and insulin resistance increase Alzheimer’s Disease (AD) risk. Individuals with these conditions, like those with AD, have demonstrated changes in the structural and functional features of the Default Mode Network (DMN). Here, we characterize associations between systemic metabolic dysfunction, brain structure (Cortical Thickness and Hippocampal Volume) and functional connectivity of DMN subnetworks.

**Method:**

We leveraged data from the Health and Aging Brain Study: Health Disparities (HABS‐HD) dataset, an ongoing longitudinal study that characterizes AD biomarkers across diverse populations. Specifically, we used data from the initial visits of the Hispanic and Non‐Hispanic White (NHW) cohorts. Individuals were categorized as cognitively healthy (n=1192; age=66.1 years, 65% female) or cognitively impaired (n=307; age=67.4 years, 50% female). We used a continuous measure of metabolic risk (Mets‐Z) that is more sensitive and ethnicity‐specific than categorical definitions of MetS. Mets‐Z a weighted composite of systolic blood pressure, triglycerides, HDL cholesterol, glucose, and waist circumference. Structural measures were acquired using FreeSurfer. Functional connectivity was measured via resting‐state fMRI, and pre‐processed using FSL. We utilized both summary metrics (ROI‐based connectivity estimations) and voxel‐wise approaches (FSL’s randomise).

**Result:**

MetS‐Z differed between diagnostic groups (t=‐2.387, p=.017) and between Hispanics and NHWs (t=‐11.864, p<.001). Meta‐ROI cortical thickness was negatively associated with both cognitive status (β = ‐.152, p < .001) and MetS‐Z (β = ‐.085, p < .001). Hippocampal volume was found to have a negative relationship with MetS‐Z (left, β = ‐.073, p < .001; right, β = ‐.072, p < .001). Functional connectivity, specifically of the posterior DMN, was significantly associated with MetS‐Z. There was a high degree of overlap between the clusters of voxels in which MetS‐Z and pDMN connectivity associated, and the clusters demonstrating decreased connectivity to due to age and cognitive status.

**Conclusion:**

We present evidence of altered DMN connectivity due to a higher load of metabolic risk in an ethnoracially diverse cohort of older adults, with and without cognitive impairment. Future studies should leverage the longitudinal HABS‐HD dataset to examine if higher metabolic dysfunction predicts differences in the trajectory of DMN connectivity.

